# An ontogenic study of receptor mechanisms by which acute administration of low-doses of methamphetamine suppresses DOI-induced 5-HT_2A_-receptor mediated head-twitch response in mice

**DOI:** 10.1186/s12868-021-00686-5

**Published:** 2022-01-04

**Authors:** Yina Sun, Seetha Chebolu, Denise Henry, Sandeep Lankireddy, Nissar A. Darmani

**Affiliations:** Department of Basic Medical Sciences, College of Osteopathic Medicine of the Pacific, Western University of Health Sciences, 309 East Second Street, Pomona, CA 91766 USA

**Keywords:** Methamphetamine, DOI, Head-twitch response, 5-HT_2A_ receptor, 5-HT_1A_ receptor, ɑ_2_-adrenergic receptor

## Abstract

**Background:**

Methamphetamine (MA) is a non-selective monoamine releaser and thus releases serotonin (5-HT), norepinephrine (NE) and dopamine (DA) from corresponding nerve terminals into synapses. DOI ((±)-2, 5-dimethoxy-4-iodoamphetamine) is a direct-acting serotonergic 5-HT_2A/C_ receptor agonist and induces the head-twitch response (HTR) via stimulation of 5-HT_2A_ receptor in mice. While more selective serotonin releasers such as d-fenfluramine evoke the HTR, monoamine reuptake blockers (e.g., cocaine) suppress the DOI-evoked HTR via indirect stimulation of serotonergic 5-HT_1A_- and adrenergic ɑ_2_-receptors. Since the induction of HTR by DOI is age-dependent, we investigated whether: (1) during development MA can evoke the HTR by itself, and (2) acute pretreatment with either the selective 5-HT_2A_ receptor antagonist EMD 281014 or low-doses of MA can: (i) modulate the DOI-induced HTR in mice across postnatal days 20, 30 and 60, and (ii) alter the DOI-induced c-*fos* expression in mice prefrontal cortex (PFC). To further explore the possible modulatory effect of MA on DOI-induced HTR, we investigated whether blockade of inhibitory serotonergic 5-HT_1A_- or adrenergic ɑ_2_-receptors by corresponding selective antagonists (WAY 100635 or RS 79948, respectively), can prevent the effect of MA on DOI-induced HTR during aging.

**Results:**

Although neither EMD 281014 nor MA by themselves could evoke the HTR, acute pretreatment with either EMD 281014 (0.01, 0.05 and 0.1 mg/kg, i.p.) or MA (1, 2.5, 5 mg/kg, i.p.), dose-dependently suppressed the DOI-induced HTR across ages. While WAY 100635 significantly reversed the inhibitory effect of MA in 20- and 30-day old mice, RS 79948 failed to significantly counter MA’s inhibitory effect. Moreover, DOI significantly increased c-*fos* expressions in several PFC regions. EMD 281014 prevented the DOI-induced increases in c-*fos* expression. Despite the inhibitory effect of MA on DOI-induced HTR, MA alone or in combination with DOI, significantly increased c-*fos* expression in several regions of the PFC.

**Conclusion:**

The suppressive effect of MA on the DOI-evoked HTR appears to be mainly due to functional interactions between the HTR-inducing 5-HT_2A_ receptor and the inhibitory 5-HT_1A_ receptor. The MA-induced increase in c-*fos* expression in different PFC regions may be due to MA-evoked increases in synaptic concentrations of 5-HT, NE and/or DA.

## Background

Methamphetamine (MA) is an amphetamine-like stimulant which are a widely-used class of illicit drugs [1, 2]. MA is clinically used for the treatment of attention hyperactivity disorder and obesity [1]. While acute effects of MA include alertness, increased energy, decreased fatigue, elevated mood, and anorexia; its prolonged abuse can result in dependence, psychosis, disturbances in mood, as well as aggression [3, 4].

MA is a psychostimulant and a non-selective monoamine releaser that promotes release of serotonin (5-HT), norepinephrine (NE) and dopamine (DA), which subsequently activate their corresponding receptors. Because of its structural similarity, MA substitutes for these monoamines at both their membrane-bound transporters, namely the serotonin transporter (SERT), NE transporter (NET) and DA transporter (DAT); as well as the vesicular monoamine transporter-2 (VMAT-2) [5, 6]. Moreover, MA also serves as a substrate for the trace amine-associated receptor 1 (TAAR1), which belongs to a family of G-protein coupled receptors that is activated by trace amines [7, 8]. It is thought MA increases monoamines synaptic concentration by: (i) redistributing monoamines from their storage vesicles into the cytosol by reversal of function of VMAT-2, and (ii) reversing the endogenous function of DAT, SERT and NET, resulting in release of 5-HT, NE, and DA from the cytosol into corresponding synapses.

Unlike human chronic MA abuse patterns, most traditional animal studies have used high-dose acute or subacute MA administration (10–40 mg/kg, one to several times a day) [9]. Such exposures lead to damage at serotonergic and dopaminergic axons and their terminals in several brain areas including the frontal cortex, striatum, and substantia nigra [5, 10]. Interestingly, MA-induced apoptosis can occur at doses less than 1 mg/kg when administered subacutely following four days of intravenous treatment [11, 12]. In general, MA-evoked brain abnormalities in animals and humans include reduced neuronal density and decreases in markers of DA, 5-HT and NE terminals such as density of DAT, SERT, NET and VMAT-2 [5, 13, 14].

The phenylalkylamine hallucinogen, DOI ((±)-2,5-dimethoxy-4-iodoamphetamine) has become one of the most common tools to study mechanisms of classical hallucinogens and the serotonergic 5-HT_2A_ receptor function [15]. Indeed, it is a high-affinity potent and selective agonist for each of the 5-HT_2A/2C_ receptor subtypes. The 5-HT_2A_-receptor-mediated head-twitch response (HTR) evoked by serotonergic hallucinogens in rodents has been considered as a potential behavioral marker for hallucinogenic effects in humans [15, 16]. In addition, systemic administration, or direct injection of DOI into the medial prefrontal cortex (mPFC), induces the HTR in rodents [17–19]. Furthermore, the HTR is not observed in 5-HT_2A_ knockout mice when administered with 5-HT_2A_ receptor agonist hallucinogens [20]. Likewise, a variety of 5-HT_2A_ receptor antagonists block the DOI-induced HTR [18]. The ontogenic development of DOI-induced HTR has been investigated and the onset of HTR is between 14 and 18 postnatal days, reaches maximal frequency at 28 days, and substantially decreases from 60 to 180 days of age [21]. Not only DOI, but also serotonin precursors (e.g., 5-hydroxytryptophan) [22] as well as selective serotonin releasers (e.g., d-fenfluramine) [23] can induce the HTR in mice.

MA binge exposure (4 × 5 mg/kg at 2 h intervals per day) has been shown to increase the frequency of DOI-induced HTR as well as the 5-HT_2A_ receptor density and expression of markers of neuronal activity (c-*fos* and Egr-2) in the mPFC of mice [24]. Furthermore, MA self-administration (males: 0.12 mg/infusion; females: 0.09 mg/infusion; 7 days) can lead to increased DOI-induced HTR in rats [25]. However, little is known about the acute effects of clinically-relevant lower acute doses of MA (0.1–5 mg/kg) on the ontogeny of DOI-induced HTR, or c-*fos* expression. In addition, although the HTR can be easily measured, it is a complex behavior that can be modulated by activation of diverse receptors [15]. Indeed, MA concomitantly increases the synaptic concentrations of 5-HT, NE and DA, and simultaneous activation of 5-HT_1A_ [26, 27]—or adrenergic ɑ_2_ [27, 28] -receptors can suppress the intensity of DOI-evoked HTRs in mice.

Thus, the initial aim of this study was to demonstrate whether varying doses of MA can induce the HTR across different ages in mice. During development MA by itself failed to induce the HTR, but it suppressed DOI-evoked HTR in a dose-dependent fashion. Subsequently, we explored the sensitivity of DOI-induced HTR across postnatal days 20, 30 and 60, to the inhibitory effects of: (i) varying doses of a new selective 5-HT_2A_ receptor antagonist EMD 281014 [29], and (ii) low doses of MA (1–5 mg/kg, i.p.). Since the ontogenic inhibitory effects of MA via the serotonergic 5-HT_1A_- or adrenergic ɑ_2_-receptors on the DOI-evoked HTR remain unknown, we utilized their corresponding selective antagonists (WAY 100635 [19] or RS 79948 [30], respectively), to see whether they can prevent the suppressive effect of MA against DOI-induced HTR across the above ages. C-*fos* has been accepted as one of the most common markers of neuronal activation in vivo [31, 32]. Administration of DOI causes induction of c-*fos* protein expression in the frontocortical and limbic brain regions [33]. In this study, we also investigated whether pretreatment with either MA or EMD 281014 [29], can alter the DOI-evoked expression of c-*fos* in a similar pattern across different regions of the PFC.

## Materials and methods

### Animals and drugs

Male albino ICR mice were used at ages of 20-, 30- and 60-days old per our previous studies [34]. The protocol was approved by the Western University of Health Sciences Institutional Animal Care and Use Committee (IACUC) and conducted with strict adherence to the recommendations in the guide for the Care and Use of Laboratory Animals of the National Institute of Health (Department of Health and Human Services Publication, revised, 2011). Mice were kept in a controlled environment (12 h light/dark cycle (light 6 am to 6 pm) and 21 ± 2 ℃ temperature) with food and water ad libitum. All efforts were made to reduce the number of animals used and to minimize their suffering.

The 5-HT_2A/2C_ receptor agonist DOI ((±)-2,5-dimethoxy-4-iodoamphetamine), the selective 5-HT_2A_ receptor antagonist EMD 281014 (7-{4-[2-(4-fluorophenyl)-ethyl]-piperazine-1-carbonyl}-1H-indole-3-carbonitrile HCl), and the selective ɑ_2_-adrenergic receptor antagonist RS 79948 (RS 79948 hydrochloride) were purchased from Tocris Bioscience (Ellisville, MO, USA). Methamphetamine (MA) and the 5-HT_1A_ receptor selective antagonist WAY 100635 (WAY 100635 maleate salt) were obtained from Sigma-Aldrich (St. Louis, MO, USA). DOI, MA, RS 79948, and WAY 100635 were dissolved in distilled water. EMD 281014 was dissolved in 0.2% tween 80 in water. All drugs were prepared on the day of the experiment and were administered via intraperitoneal (i.p.) injection at a volume of 0.1 ml/10 g of body weight.

### Behavioral experiments

On the day of experiments, mice were brought from the animal facility and randomly assigned to vehicle-treated control and treatment groups. Animals were separated into individual cages and allowed to adapt to the experimentation room for at least 2 h. The HTR is a very distinctive head-twitching behavior in mice and usually cannot be mistaken for such behaviors as head shakes (lateral movement of the head from side to side) or head jerks (up and down jerking) [35].

**Fig. 1:**
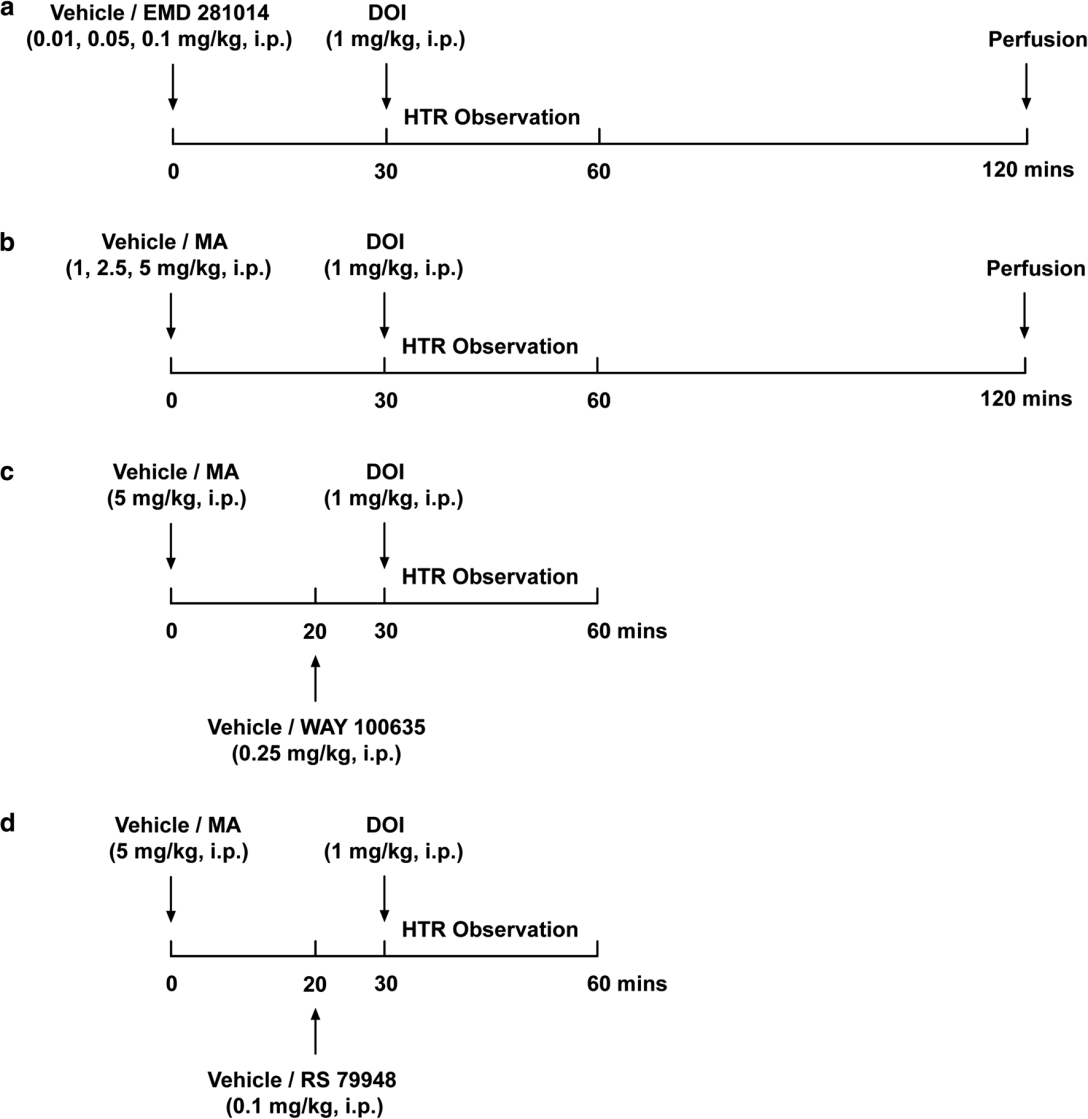
Timelines for injections, HTR observation, and perfusion. **a** and **b** Corresponding vehicle (i.p.), or varying doses of the EMD 281014 (0.01, 0.05, 0.1 mg/kg, i.p.), or MA (1, 2.5, 5 mg/kg, i.p.), were injected to different groups of mice (20-, 30- or 60-day old) 30 min prior to DOI (1 mg/kg, i.p.) injection. The frequency of HTRs were observed for 30 min post DOI injection. Based on behavioral data, 30-day-old mice were selected to perform the immuno-histochemistry studies. Thus, after injections and HTR observation, mice were perfused at 120 min (**a**, **b**). For behavioral interaction studies, different groups of mice (20-, 30, and 60-day old) received either a single dose of MA (5 mg/kg, i.p.) or its vehicle at 0 min, and at 20 min received either the 5-HT_1A_ receptor antagonist WAY 100635 (0.25 mg/kg, i.p.), or the ɑ_2_-adrenergic-receptor antagonist RS 79948 (0.1 mg/kg, i.p.), or corresponding vehicle. At 30 min DOI (1 mg/kg, i.p.) was injected (**c**, **d**) and the HTR frequency were observed for 30 min post DOI injection

Based upon our preliminary and published studies [26, 36, 37], we investigated the effect of the selective 5-HT_2A_ receptor antagonist EMD 281014 (a positive control) or MA, on the DOI-induced HTR in 20-, 30- and 60-day old mice. Thus, at 0 min different groups of mice were pretreated with an injection of either the corresponding vehicle (i.p.), or varying doses of the selective 5-HT_2A_ receptor antagonist EMD 281014 (0.01, 0.05, 0.1 mg/kg, i.p.) or MA (1, 2.5, 5 mg/kg, i.p.). Thirty minutes later, each treated mouse received a 1 mg/kg dose of DOI [35] (i.p.; Fig. 1a, b). According to our preliminary and published studies [26–28], we evaluated whether blockade of serotonergic 5-HT_1A_- or ɑ_2_-adrenergic-receptor could affect the suppressive effect of MA (5 mg/kg, i.p.) on the DOI-induced HTR, different groups of mice were injected with either vehicle (i.p.) or a single dose of MA (5 mg/kg, i.p.) at 0 min. Twenty min later, these mice were pretreated with either WAY 100635 (0.25 mg/kg, i.p.) or RS 79948 (0.1 mg/kg, i.p.). At 30 min, the treated mice received an injection of DOI (1 mg/kg, i.p.) (Fig. 1c, d). Each mouse was individually observed immediately following the injection of DOI and the HTR score (mean ± SEM) was recorded cumulatively at 5-min intervals for the next 30 min [27]. The observer was blind to animals’ treatment conditions. Each animal was used once and then euthanized with isoflurane (3%) after the termination of each experiment. All behavioral experiments were conducted between 9:00 am and 4:00 pm.

### Immunohistochemistry

In order to observe whether pretreatment with either MA or EMD 281014 would alter the expressions of c-*fos* evoked by DOI (1 mg/kg, i.p.) across different regions in the PFC, based on behavioral data we chose 30 days old mice pretreated with MA (5 mg/kg, i.p.) or EMD 281014 (0.1 mg/kg, i.p.) to perform our immunohistochemistry studies in accord with our experimental design in Fig. 1a, b.

Two hours after the first injection, mice were deeply anesthetized with isoflurane (3%) and were then transcardially perfused with 0.01 M phosphate buffered saline (PBS, VWR) followed by 4% paraformaldehyde (J. T. Baker). The brains were removed immediately and post-fixed in the same fixative for 2 h, and then placed in 0.1 M PB containing 30% sucrose at 4℃ until they sank. The coronal frozen sections of the PFC were cut at 25 µm sections using a cryostat. After pre-incubating with 10% normal donkey serum and 0.3% Triton X-100 in PBS for 1 h at room temperature, the sections were incubated in a rabbit polyclonal anti-c-*fos* primary antibody (1:1000; Abcam, Cambridge, UK) diluted in the primary antibody dilution (0.01 M PBS containing 5% normal donkey serum, 0.05% sodium azide, and 0.3% Triton X-100) at 4 ℃ overnight. Thereafter, they were washed in PBS 3 times, and were then incubated with Alexa 594-conjugated goat anti-rabbit secondary antibody (1:1000; Invitrogen) diluted in the secondary antibody dilution (0.01 M PBS containing 0.3% Triton X-100) for 4 h at room temperature. After washing several times, the sections were mounted and coverslipped with anti-fade mounting medium containing DAPI (Vector Laboratories). The experimenter acquiring and analyzing the images were blind to experimental condition.

### Image analysis

**Fig. 2:**
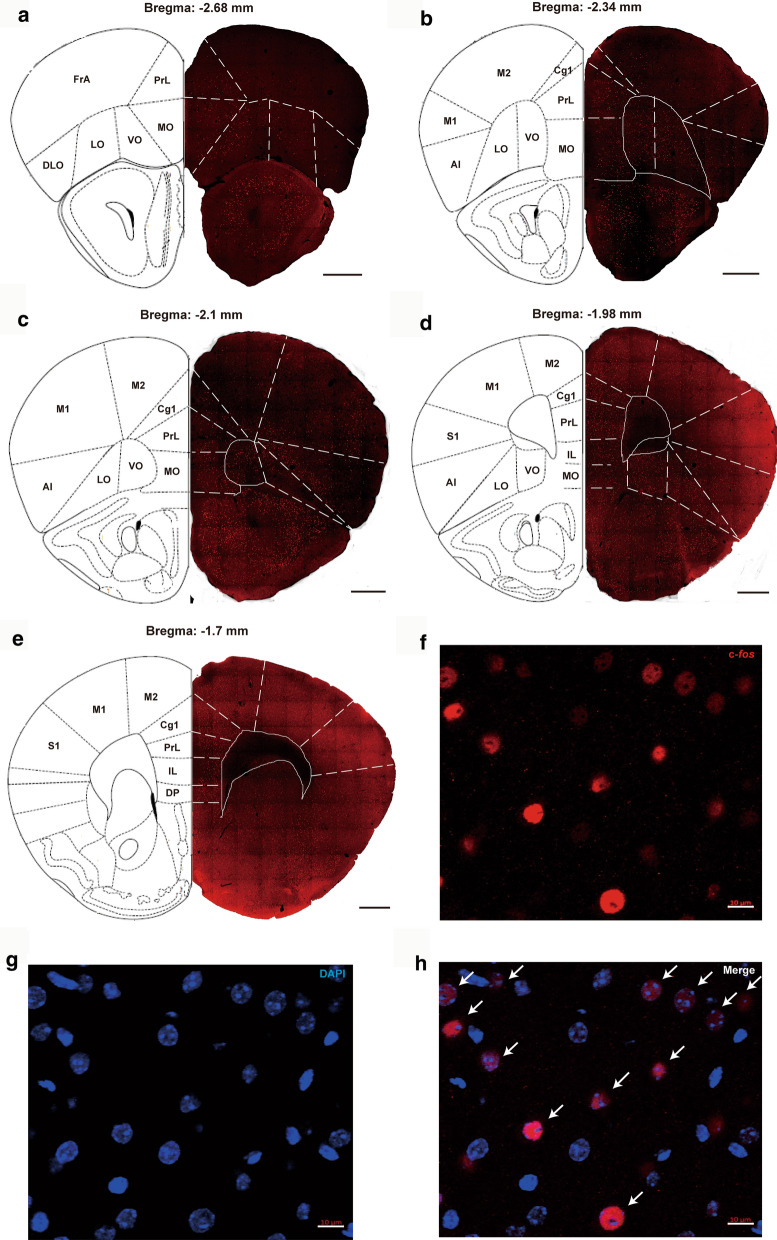
**a**−**e** show different regions of the PFC sections of the vehicle + DOI treated mice brain at 5 coronal levels (− 2.68 mm, − 2.34 mm, − 2.1 mm, − 1.9 mm, and − 1.7 mm relative to bregma Paxinos and Franklin [38]), in which c-*fos* numbers were counted, scale bars = 500 μm (**a**−**e**). Illustrations are adapted from the atlas of Paxinos and Franklin [38]. c-*fos* immunoreactivity was counted when the cell nucleus was round or oval, completely filled and double-labeled with DAPI, scale bars = 10 μm (**f**−**h**). Abbreviations: AI: agranular insular cortex; Cg1: cingulate cortex area 1; DLO: dorsal lateral orbital cortex; DP: dorsal peduncular cortex; FrA: frontal associated cortex; IL: infralimbic cortex; LO: lateral orbital cortex; M1: primary motor cortex; M2: secondary motor cortex; MO: medial orbital cortex; PrL: prelimbic cortex, S1: primary somatosensory area; VO: ventral orbital cortex

Images were acquired using a Zeiss LSM 880 confocal laser-scanning microscope and were captured at 20× and 60× magnification. Images for all groups in a given experiment were obtained using identical acquisition parameters and analyzed using ImageJ software (NIH). In each mouse brain, c-*fos* expressions in different regions of three consecutive sections at 5 coronal levels (− 2.68 mm, − 2.34 mm, − 2.1 mm, − 1.98 mm, and − 1.7 mm relative to bregma [38], Fig. 2a–e) in the PFC were analyzed. The numbers of c-*fos* in the following regions of each coronal level were counted, in the section of bregma − 2.68 mm: frontal associated cortex (FrA), prelimbic cortex (PrL), medial orbital cortex (MO), ventral orbital cortex (VO), lateral orbital cortex (LO), dorsal lateral orbital cortex (DLO); in the sections of bregma − 2.34 mm and − 2.1 mm: primary motor cortex (M1), secondary motor cortex (M2), cingulate cortex area 1 (Cg1), PrL, MO, VO, LO, agranular insular cortex (AI); in the section of bregma − 1.98 mm: primary somatosensory area (S1), M1, M2, Cg1, PrL, infralimbic cortex (IL), MO, VO, LO, AI; in the section of bregma − 1.7 mm: S1, M1, M2, Cg1, PrL, IL, dorsal peduncular cortex (DP). c-*fos* immunoreactivity was counted when the cell nucleus was round or oval, completely filled and double-labeled with DAPI (Fig. 2f–h). The number of c-*fos* in each area was calculated from the average of the numbers from three consecutive sections for each mouse brain. The values from 5 to 6 mice of each treatment group were averaged to obtain the final mean ± SEM.

### Statistical analysis

Statistical analyses were performed using the Graphpad Prism 8 (Graphpad software Inc., San Diego, CA). Behavioral data were analyzed by two-way analysis of variance (ANOVA) followed by Bonferroni’s test for multiple comparisons when F-test was significant. Histological data were analyzed by one-way ANOVA followed by Tukey's multiple test. All data were expressed as mean ± SEM. A *p* value of less than 0.05 was considered significant.

## Results

### DOI-induced HTR was dose-dependently blocked by the selective 5-HT_2A_ receptor antagonist EMD 281014 across the age-range tested

**Fig. 3:**
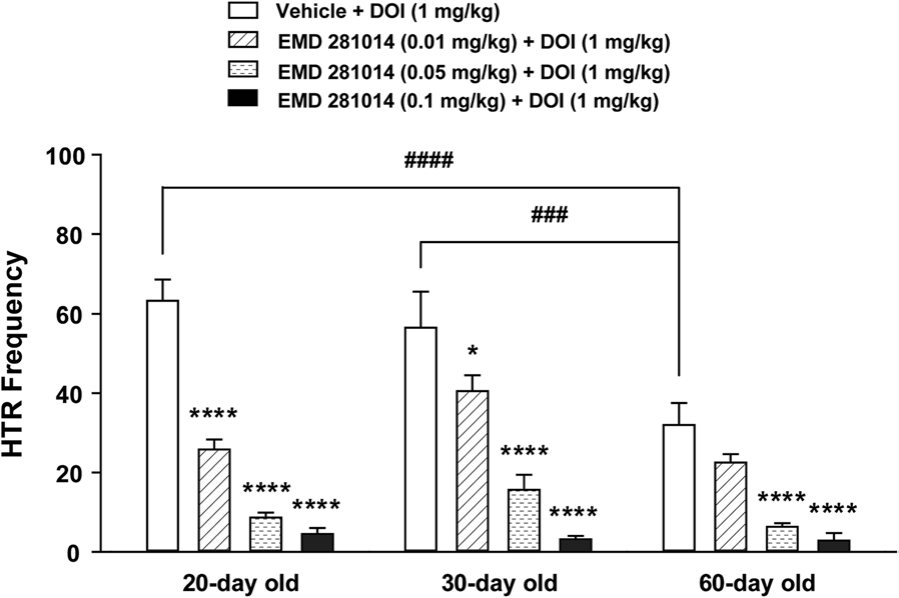
Suppressive effects of varying doses of the selective 5-HT_2A_ receptor antagonist EMD 281014 (0, 0.01, 0.05 and 0.1 mg/kg, i.p.) on the frequency of HTR induced by DOI (1 mg/kg, i.p.) across different ages (20-, 30- and 60-day old) in mice. In EMD 281014 vehicle-pretreated control mice, the mean frequency of DOI-induced HTR tended to gradually decrease with increasing age, with significant differences occurring between 20- and 60-day (^####^*p* < 0.0001) and between 30- and 60-day old mice (^###^*p* = 0.0002). Varying doses of EMD 281014 suppressed DOI-induced HTR in a dose- and age-dependent manner across different ages. Compared to corresponding age-matched vehicle-pretreated control group, significant reductions were observed at all tested doses of EMD 281014 in 20- and 30-day old mice, but only at 0.05 and 0.1 mg/kg doses in 60-day old mice. *p = 0.0181, ****p < 0.0001 vs. vehicle injection; two-way ANOVA followed by Bonferroni's test. *n* = 6 in each group. Data are presented as means ± SEM.

Previous research has demonstrated that the frequency of DOI-induced HTR in mice gradually decreases during aging [21]. In the current study we confirm this finding (Fig. 3). Administration of varying doses of the 5-HT_2A_ receptor antagonist EMD 281014 (0.01, 0.05 and 0.1 mg/kg, i.p.) by itself had no effect on basal HTR scores in 20-, 30-, and 60-days old mice (all 0 ± 0; *n* = 6 per group). A two-way ANOVA (age × dose of drug) showed significant effects on the frequency of DOI-induced HTR for age (*F*_*2, 60*_ = 11.59, *p* < 0.0001), dose of EMD 281014 (*F*_*3, 60*_ = 85.24, *p* < 0.0001), and their interaction (*F*_*6, 60*_ = 4.275, *p* = 0.0012; Fig. 3). The frequency of DOI-induced HTR in vehicle-pretreated mice tended to gradually decrease with increasing age, with significant differences occurring between 20- and 60-day (*p* < 0.0001, *n* = 6 mice per group), and between 30- and 60-day old mice (*p* = 0.0002, *n* = 6 mice per group; Bonferroni's test; Fig. 3). Relative to the corresponding age-matched vehicle-pretreated control group, varying doses of EMD 281014 (0.01, 0.05 and 0.1 mg/kg, i.p., *n* = 6 mice per group) suppressed DOI-induced HTR in a dose-dependent manner across the age-range tested. Indeed, in 20- and 30-day old mice, all tested doses of EMD 281014 significantly decreased the frequency of DOI-induced HTR (*p* = 0.0181 for EMD 281014 at 0.01 mg/kg in 30-day-old mice, all other *p* < 0.0001; Bonferroni's test; Fig. 3). In 60-day old mice, significant decreases were only observed at its 0.05 and 0.1 mg/kg doses (both *p* < 0.0001; Bonferroni's test; Fig. 3).

### DOI-induced HTR was dose-dependently suppressed by MA across the age-range tested

**Fig. 4:**
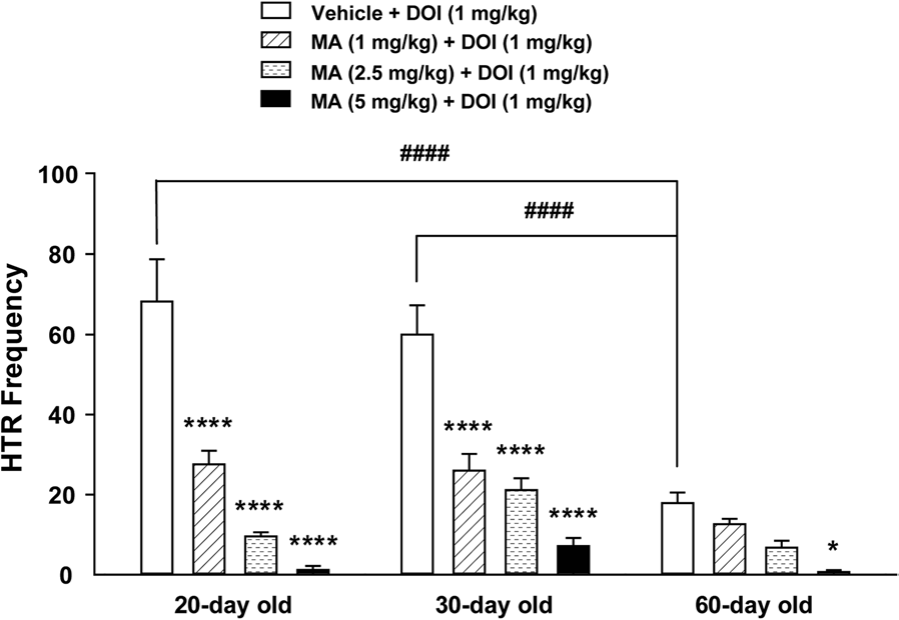
Suppressive effects of varying doses of MA (0, 1, 2.5 5 mg/kg, i.p.) on the frequency of HTR induced by DOI (1 mg/kg, i.p.) across different ages (20-, 30- and 60-day old) in mice. In the MA vehicle-pretreated control group, the frequencies of DOI-induced HTR gradually decreased with increasing age. Significant differences were found between 20- and 60-day (^####^*p* < 0.0001), and between 30- and 60-day old mice (^####^*p* < 0.0001). Relative to the corresponding age-matched vehicle-pretreated controls, MA inhibited the mean frequency of DOI-induced HTRs across different ages in a dose- and age-dependent manner. Significant differences were observed at all tested doses of MA in 20- and 30-day old mice, whereas the significant effect was only observed at 5 mg/kg of MA in 60-day old mice. *p = 0.0432*,* ****p < 0.0001 vs. vehicle injection; two-way ANOVA followed by Bonferroni's test. *n* = 5−8 in each group. Data are presented as means ± SEM.

Administration of MA (1, 2.5, 5 mg/kg, i.p.) by itself had no effect on basal HTR frequency in 20-, 30-, and 60-day old mice (all 0 ± 0; *n* = 6 per group). We also examined whether systemic administration of MA (0, 1, 2.5, 5 mg/kg) could modulate the frequency of DOI-induced HTR across different ages. A two-way ANOVA (age × dose of MA) showed significant effects of age (*F*_*2, 70*_ = 17.83, *p* < 0.0001), dose of MA (*F*_*3*__*70*_ = 53.19, *p* < 0.0001), and age × dose of drug interaction (*F*_*6,*__*70*_ = 6.508, *p* < 0.0001; Fig. 4). The frequency of DOI-induced HTR in MA vehicle-pretreated mice gradually decreased with increasing age with significant differences between 20- and 60-day (*p* < 0.0001) and between 30- and 60-day old mice (*p* < 0.0001, *n* = 8 mice per age group; Bonferroni's test; Fig. 4). Relative to the corresponding age-matched vehicle-pretreated control group, MA at all tested doses significantly suppressed the frequency of DOI-induced HTR in 20- and 30-day mice (all *p* < 0.0001, *n* = 6**−**7 mice per group; Bonferroni's test), while in 60-day old mice MA could only significantly suppress the frequency of DOI-evoked HTR at its 5 mg/kg dose. (*p* = 0.0432, *n* = 5 mice; Bonferroni's test; Fig. 4).

### Blockade of 5-HT_1A_ receptor significantly reversed the inhibitory effect of MA on DOI-induced HTR across the age-range tested

**Fig. 5:**
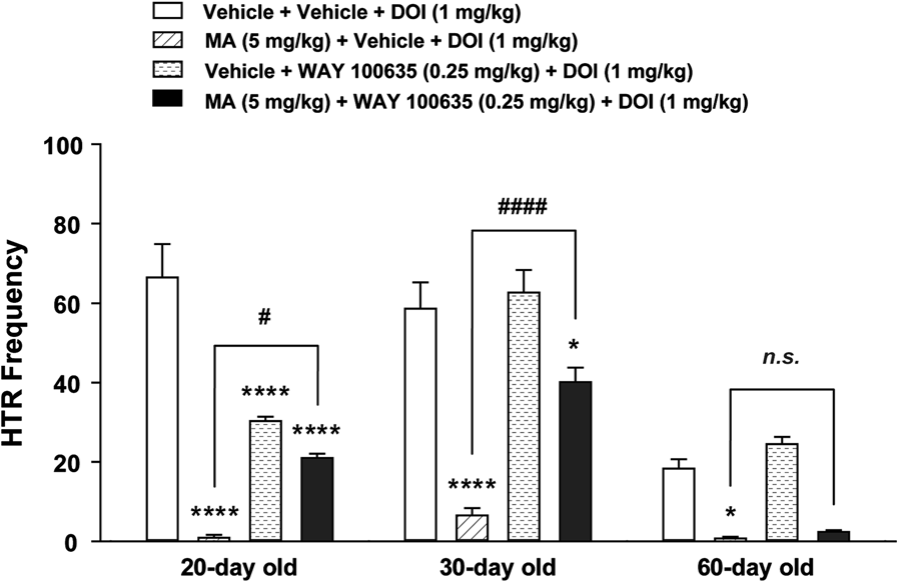
Reversal effect of the selective 5-HT_1A_ receptor antagonist WAY 100635 (0.25 mg/kg, i.p.) on the inhibitory action of MA (5 mg/kg, i.p.) on DOI-induced (1 mg/kg, i.p.) HTR across different ages (20-, 30-, and 60-day old) in mice. Relative to the corresponding age-matched Vehicle + Vehicle + DOI pretreated control group, MA pretreatment (i.e. MA + Vehicle + DOI treatment group) significantly reduced the frequency of DOI-induced HTR in 20- 30- (all ****p < 0.0001) and 60-day old mice (*p = 0.0404). WAY 100635 by itself (i.e. in the Vehicle + WAY 100635+ DOI group) markedly attenuated the frequency of DOI-induced HTR in 20-day mice but did not affect the evoked behavior in 30- or 60-day old mice. Relative to the corresponding MA + Vehicle + DOI treatment control group at each age group, inclusion of the 5-HT_1A_ receptor antagonist WAY 100635 (i.e. the MA + WAY 100635 + DOI treatment group) reversed the inhibitory effect of MA on DOI-induced HTR across all ages but significance was observed only during days 20 and 30. ^#^*p* = 0.0239, ^####^*p* < 0.0001 vs. MA + Vehicle + DOI group; two-way ANOVA followed by Bonferroni's test. *n* = 6 − 10 in each group. Data are presented as means ± SEM.

We have previously shown that while the selective 5-HT_1A_ receptor agonist 8-OH-DPAT suppresses the frequency of DOI-evoked HTRs [26], the selective 5-HT_1A_ receptor antagonist WAY 100635 can reverse this effect [19]. In the present study a two-way analysis of ANOVA (age × treatment) showed highly significant differences of age (*F*_*2, 78*_ = 43.43, *p* < 0.0001), treatment (*F*_*3, 78*_ = 61.01, *p* < 0.0001), and age × treatment interaction (*F*_*6, 78*_ = 8.706, *p* < 0.0001; Fig. 5). Indeed, relative to the corresponding age-matched vehicle-pretreated control group (i.e. vehicle + vehicle + DOI, *n* = 9**−**10 mice per age group), MA (i.e. the MA + Vehicle + DOI group, *n* = 7**−**8 mice per age group) significantly decreased the frequency of DOI-induced HTR in 20-, 30- and 60-day mice (*p* < 0.0001 for 20- and 30-day mice, *p* = 0.0404 for 60-day mice; Bonferroni's test; Fig. 5). Inclusion of the 5-HT_1A_ antagonist WAY 100635 (i.e. the MA + WAY 100635 + DOI treatment group, *n* = 6**−**8 mice per age group) reversed the inhibitory effect of MA on DOI-induced HTR across all ages, but significance was observed only in 20 (*p* = 0.0239)- and 30 (*p* < 0.0001; Bonferroni's test; Fig. 5)-day old mice. WAY 100635 by itself (i.e. in the Vehicle + WAY 100635 + DOI group, *n* = 6**−**7 mice per age group) markedly attenuated the frequency of DOI-induced HTR in 20-day mice but did not affect the evoked behavior in 30- or 60-day old mice.

### The effect of blockade of ɑ_2_-adrenergic receptor on the inhibitory action of MA on DOI-induced HTR

**Fig. 6:**
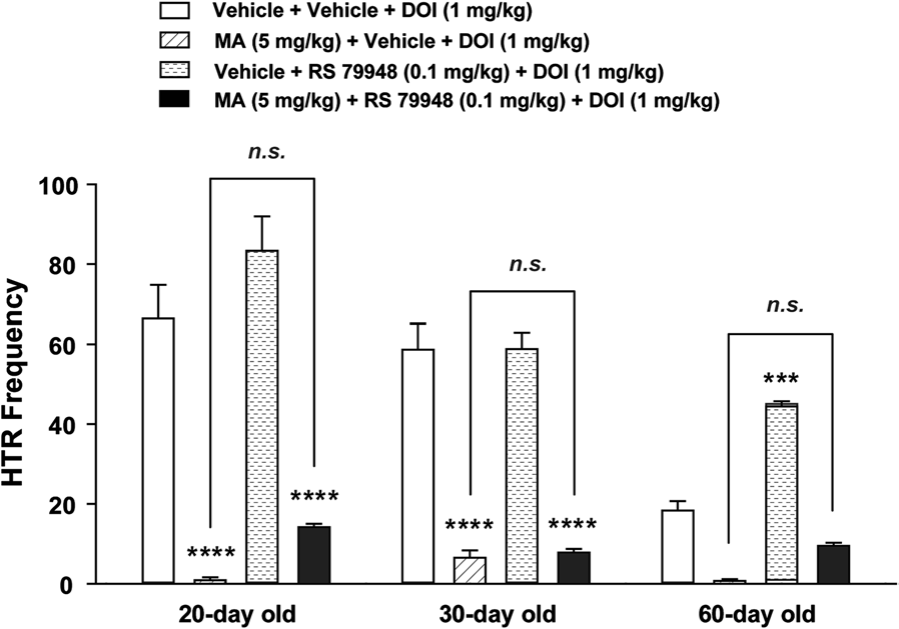
Effects of the selective ɑ_2_-adrenergic receptor antagonist RS 79948 (0.25 mg/kg, i.p.) on the inhibitory action of MA (5 mg/kg, i.p.) on the frequency of HTR induced by DOI (1 mg/kg, i.p.) across different ages (20-, 30- and 60-day old) in mice. Compared to the corresponding Vehicle + Vehicle + DOI treatment control group at each age group, inclusion of MA (i.e. MA + Vehicle + DOI treatment group) significantly decreased the mean frequency of HTR induced by DOI in 20- and 30-day old mice, but no significant effect was seen in 60-day old mice. RS 79948 pretreatment tended to reverse the inhibitory effect of MA (i.e. MA + RS 79948 + DOI treatment group) across different ages but the effect failed to attain significance. ***p = 0.001, ****p < 0.0001 vs. Vehicle + Vehicle + DOI group. *n.s.*: no significant difference *vs*. MA + Vehicle + DOI group; two-way ANOVA followed by Bonferroni's test. *n* = 5 − 10 in each group. Data are presented as means ± SEM.

We have previously shown that the ɑ_2_-adrenergic receptor antagonist yohimbine prevents the inhibitory effects of the monoamine reuptake blocker cocaine on DOI-induced HTR [27]. In the current study a two-way analysis of ANOVA (age × treatment) showed highly significant differences among the ages (*F*_*2, 75*_ = 22.7, *p* < 0.0001), treatment (*F*_*3, 75*_ = 100.3, *p* < 0.0001), and age × treatment interaction (*F*_*6, 75*_ = 7.919, *p* < 0.0001; Fig. 6). Indeed, relative to the corresponding age-matched vehicle-pretreated control group (i.e. vehicle + vehicle + DOI, *n* = 9**−**10 mice per age group), MA (5 mg/kg, i.p.) pretreatment (i.e. MA + Vehicle + DOI treatment group) significantly decreased the mean frequency of DOI-induced HTR in 20- and 30-day (both *p* < 0.0001, *n* = 7 mice per age group; Bonferroni's test; Fig. 6), but not in 60-day-old mice (*n* = 8 mice; Fig. 6). RS 79948 pretreatment did not significantly reverse the inhibitory effect of MA on DOI-induced HTR across different ages (*n* = 5** − **7 mice per age group; Fig. 6). RS 79948 by itself (i.e. in the Vehicle + RS 79948 + DOI group, *n* = 6 mice per age group) significantly increased the frequency of DOI-induced HTR in 60-day mice but did not affect the evoked behavior in 20- or 30-day old mice.

### DOI-induced c-fos expression in the PFC was prevented by pretreatment with EMD 281014

**Fig. 7:**
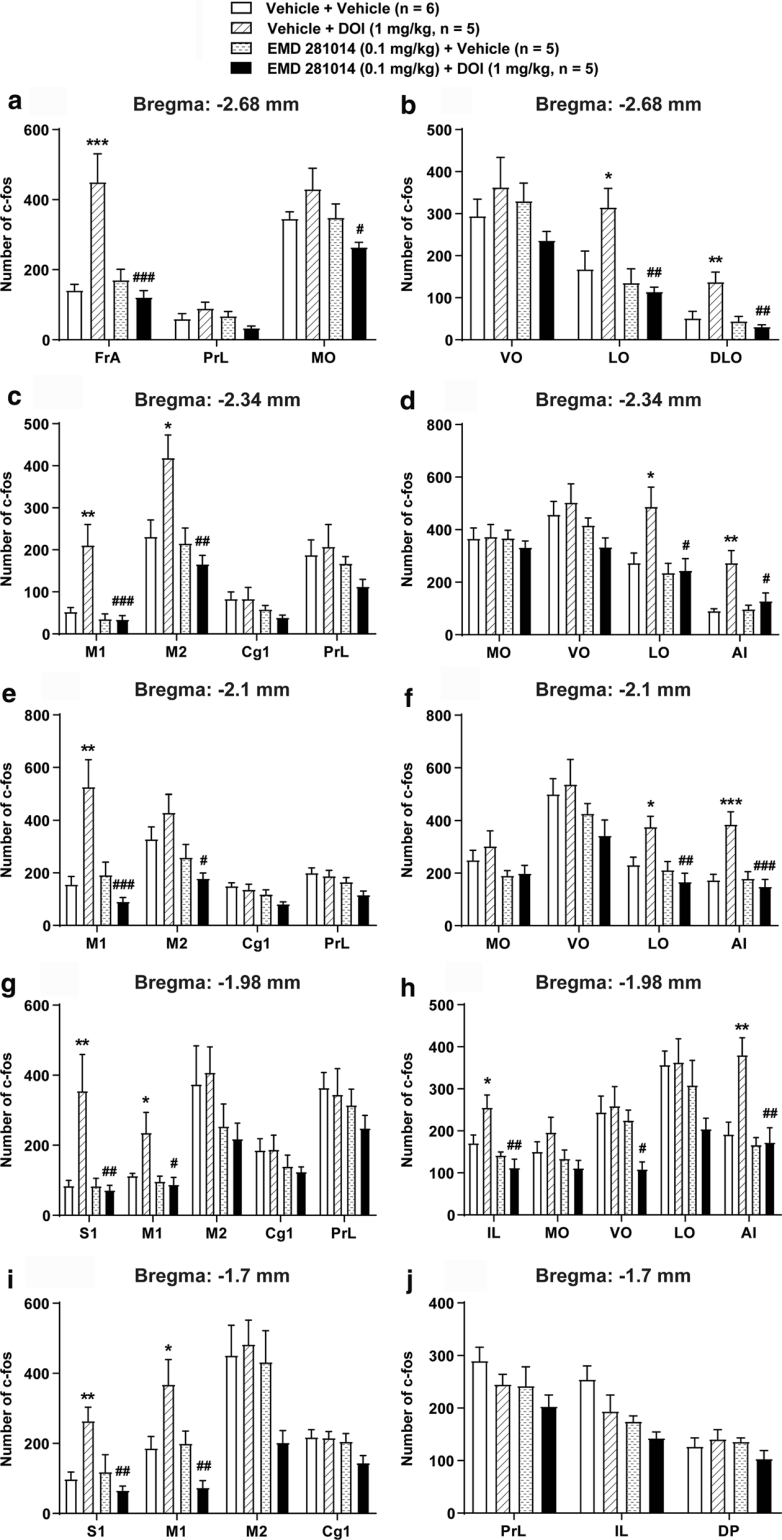
Suppressive effects of the selective 5-HT_2A_ receptor antagonist EMD 281014 on DOI-induced c-*fos* expression in different regions at 5 coronal sections in the PFC. Compared to the corresponding vehicle + vehicle pretreated control-mice, administration of DOI (i.e. vehicle + DOI (1 mg/kg, i.p.)) evoked greater numbers of c-*fos* positive cells in the: (i) FrA, LO, and DLO at the level of − 2.68 mm relative to bregma (**a**, **b**); (ii) M1, M2, LO, and AI at the level of − 2.34 mm relative to bregma (**c**, **d**); (iii) M1, LO, and AI at the level of − 2.1 mm relative to bregma (**e**, **f**); (iv) S1, M1, IL, and AI at the level of − 1.98 mm relative to bregma (**g**, **h**) and (v) S1 and M1 at the level of − 1.7 mm relative to bregma (**i**, **j**). Pretreatment with the selective 5-HT_2A_ receptor antagonist EMD 281014 (0.1 mg/kg, i.p.) prevented the DOI-induced c-*fos* expression in the: (i) FrA, MO, LO, and DLO at the level of − 2.68 mm relative to bregma (**a**, **b**); (ii) M1, M2, LO, and AI at the level of − 2.34 mm relative to bregma (**c**, **d**); (iii) M1, M2, LO, and AI at the level of − 2.1 mm relative to bregma (**e**, **f**); (iv) S1, M1, IL, VO, and AI at the level of − 1.98 mm relative to bregma (**g**, **h**); and (v) S1 and M1 at the level of − 1.7 mm relative to bregma (**i**, **j**). Treatment with EMD 281014 by itself (i.e. EMD 281014 + vehicle group) did not produce a change in c-*fos* expression in any of the regions tested when compared to vehicle + vehicle treatment control group. *p < 0.05, **p < 0.01, ***p < 0.001 vs. Vehicle + Vehicle pretreated control-mice. ^#^*p* < 0.05, ^##^*p* < 0.01 ^###^*p* < 0.001 vs*.* Vehicle + DOI treatment group one-way ANOVA followed by Tukey's test. Data are presented as means ± SEM

In the present study, we determined the levels of DOI-induced c*-fos* expressions under various experimental conditions in different regions of the PFC sections of each mouse brain at 5 coronal levels [38]. In vehicle + vehicle pretreated control-mice (*n* = 6), mild basal levels of c-*fos* immunoreactivity were observed in different regions of the PFC sections (Fig. 7). Compared to this control group, DOI (i.e. Vehicle + DOI, *n* = 5) induced a greater number of c-*fos* positive cells in the: (i) FrA (*p* = 0.0004), LO (*p* = 0.0491), and DLO (*p* = 0.0056) at the level of − 2.68 mm relative to bregma (Fig. 7a, b); (ii) M1 (*p* = 0.0016), M2 (*p* = 0.0172), LO (*p* = 0.0307), and AI (*p* = 0.0012) at the level of − 2.34 mm relative to bregma (Fig. 7c, d); (iii) in the M1 (*p* = 0.0013), LO (*p* = 0.0322), and AI (*p* = 0.001) at the level of − 2.1 mm relative to bregma (Fig. 7e, f); (iv) S1 (*p* = 0.0076), M1 (*p* = 0.0444), IL (*p* = 0.0438), and AI (*p* = 0.0025) at the level of − 1.98 mm relative to bregma (Fig. 7g, h); and (v) S1 (*p* = 0.0095) and M1 (*p* = 0.0378) at the level of − 1.7 mm relative to bregma (Fig. 7i, j).

The 5-HT_2A_ receptor antagonist EMD 281014 (0.1 mg/kg, i.p., *n* = 5) significantly prevented the DOI-induced c-*fos* expression in the: (i) FrA (*p* = 0.0003), MO (*p* = 0.0291), LO (*p* = 0.0082), and DLO (*p* = 0.0012) at the level of − 2.68 mm relative to bregma (Fig. 7a, b); (ii) M1 (*p* = 0.0008), M2 (*p* = 0.0022), LO (*p* = 0.0175), and AI (*p* = 0.012) at the level of − 2.34 mm relative to bregma (Fig. 7c, d); (iii) M1 (*p* = 0.0004), M2 (*p* = 0.0143), LO (*p* = 0.0028), and AI (*p* = 0.0005) at the level of − 2.1 mm relative to bregma (Fig. 7e, f); (iv) S1 (*p* = 0.0075), M1 (*p* = 0.0183), IL (*p* = 0.001), VO (*p* = 0.0358), and AI (*p* = 0.0016) at the level of − 1.98 mm relative to bregma (Fig. 7g, h); and (v) S1 (*p* = 0.0032) and M1 (*p* = 0.0012) at the level of − 1.7 mm relative to bregma (Fig. 7i, j). EMD 281014 (0.1 mg/kg, i.p.) by itself (i.e. EMD 281014 (0.1 mg/kg, i.p.) + vehicle group, *n* = 5) did not alter c-*fos* expressions in diverse regions of the PFC compared to vehicle + vehicle control group. These data demonstrate that DOI-evoked increases in c-*fos* expressions in diverse regions of the PFC occurs via 5-HT_2A_ receptors.

### MA by itself increases but does not affect DOI-induced c-fos expression in the PFC

**Fig. 8:**
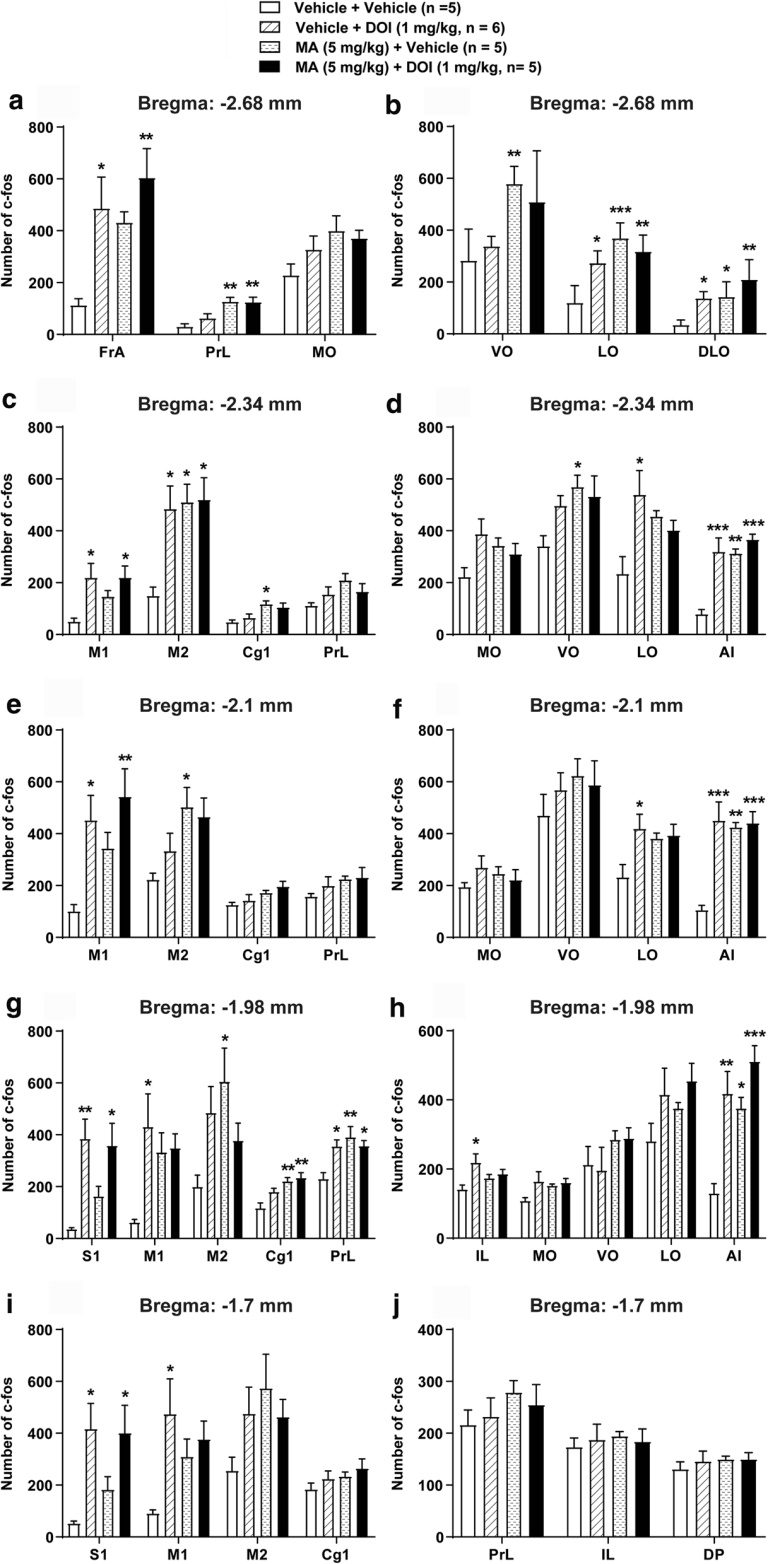
Effects of DOI and MA administration either alone or in combination on c-*fos* expression in different regions at 5 coronal levels in the PFC. Relative to the corresponding vehicle + vehicle treatment control group, DOI by itself (i.e. vehicle + DOI (1 mg/kg, i.p.)) significantly increased c-*fos* expression in the: (i) FrA, LO, and DLO at the level of − 2.68 mm relative to bregma (**a**, **b**); (ii) M1, M2, LO, and AI at the level of − 2.34 mm relative to bregma (**c**, **d**); (iii) M1, LO, and AI at the level of − 2.1 mm relative to bregma (**e**, **f**); (iv) S1, M1, PrL, IL, and AI at the level of − 1.98 mm relative to bregma (**g**, **h**) and (v) S1 and M1 at the level of − 1.7 mm relative to bregma (**i**, **j**). MA by itself (i.e. MA + vehicle group) significantly increased the expressions of c-*fos* in the: (i) PrL, VO, LO, and DLO at the level of − 2.68 mm relative to bregma (**a**, **b**); (ii) M2, Cg1, VO, and AI at the level of − 2.34 mm relative to bregma (**c**, **d**); (iii) M2, and AI at the level of − 2.1 mm relative to bregma (**e**, **f**). (iv) M2, Cg1, PrL, and AI at the level of − 1.98 mm relative to bregma (**g**, **h**). Combined treatment with MA + DOI significantly increased the expressions of c-*fos* in the: (i) FrA, PrL, LO, and DLO at the level of − 2.68 mm relative to bregma (**a**, **b**); (ii) M1, M2, and AI at the level of − 2.34 mm relative to bregma (**c**, **d**); (iii) M1, and AI at the level of − 2.1 mm relative to bregma (**e**, **f**); (iv) S1, Cg1, PrL and AI at the level of − 1.98 mm relative to bregma (**g**, **h**); and (v) S1 at the level of − 1.7 mm relative to bregma (**i**, **j**). There are no significant differences among comparisons of vehicle + DOI vs*.* MA + vehicle; vehicle + DOI vs. MA + DOI; and MA + vehicle vs. MA + DOI in the c-*fos* expressions in these brain regions. *p < 0.05, **p < 0.01, ***p < 0.001 vs. Vehicle + Vehicle treatment control group; one-way ANOVA followed by Tukey's test. Data are presented as means ± SEM

DOI in MA-vehicle pretreated mice evoked a similar c*-fos* expression in diverse regions of the PFC (*n* = 6; Fig. 8) to that already described for the EMD-vehicle treatment group (Fig. 7). Compared to vehicle + vehicle control group (*n* = 5), treatment with MA by itself (MA + vehicle group, *n* = 5) significantly increased the expressions of c-*fos* in the: (i) PrL (*p* = 0.0044), VO (*p* = 0.0098), LO (*p* = 0.0009), and DLO (*p* = 0.0453) at the level of − 2.68 mm relative to bregma (Fig. 8a, b); (ii) M2 (*p* = 0.0193), Cg1 (*p* = 0.0141), VO (*p* = 0.0379), and AI (*p* = 0.0011) at the level of − 2.34 mm relative to bregma (Fig. 8c, d); (iii) M2 (*p* = 0.0391), and AI (*p* = 0.0016) at the level of − 2.1 mm relative to bregma (Fig. 8e, f); and (iv) M2 (*p* = 0.0371), Cg1 (*p* = 0.0035), PrL (*p* = 0.0068), and AI (*p* = 0.0121) at the level of − 1.98 mm relative to bregma (Fig. 8g, h).

Relative to vehicle + vehicle treatment group, treatment with MA + DOI (*n* = 5) also significantly increased the expressions of c-*fos* in the: (i) FrA (*p* = 0.0086), PrL (*p* = 0.0055), LO (*p* = 0.0074), and DLO (*p* = 0.0011) at the level of − 2.68 mm relative to bregma (Fig. 8a, b); (ii) M1 (*p* = 0.047), M2 (*p* = 0.0162), and AI (*p* = 0.0001) at the level of − 2.34 mm relative to bregma (Fig. 8c, d); (iii) M1 (*p* = 0.0084), and AI (*p* = 0.001) at the level of − 2.1 mm relative to bregma (Fig. 8e, f); (iv) S1 (*p* = 0.0127), Cg1 (*p* = 0.0011), PrL (*p* = 0.0362), and AI (*p* = 0.0002) at the level of − 1.98 mm relative to bregma (Fig. 8g, h); and (v) S1 (*p* = 0.0369) at the level of − 1.7 mm relative to bregma (Fig. 8i, j). There are no significant differences among comparisons of vehicle + DOI vs. MA + vehicle; vehicle + DOI vs. MA + DOI; and MA + vehicle *vs.* MA + DOI in the c-*fos* expressions in these brain regions. These data indicate that administration of MA dramatically increases c-*fos* expressions in several regions of the PFC.

## Discussion

It is well established that low doses of MA can increase the synaptic concentration of monoamines (5-HT, NE, and DA) in the PFC [39]. The current study suggests that during development low doses of MA (1–5 mg/kg, i.p.) can suppress the ability of the direct-acting 5-HT_2A/C_ receptor agonist DOI to evoke the HTR in mice in a dose-dependent manner across the age-range tested. Since MA lacks direct affinity for 5-HT_2A_, 5-HT_1A_—or adrenergic ɑ_2_-receptors [40], its suppressive effect on the HTR appears to be mainly due to indirect activation of the inhibitory serotonergic 5-HT_1A_- and to a lesser degree adrenergic ɑ_2_- receptors. In fact, the selective 5-HT_1A_ receptor antagonist WAY 100635 significantly reversed the inhibitory effect of MA on the DOI-induced HTR, whereas the selective ɑ_2_-adrenergic receptor antagonist RS 79948 failed to do so. This indirect suppressive effect of MA is further reflected by ability of the selective 5-HT_2A_-receptor antagonist EMD 281014 to suppress both DOI-evoked HTR and corresponding c-*fos* immunoreactivity in several regions of the PFC, whereas MA attenuated the DOI-evoked HTR, but not the evoked c-*fos* expression.

### DOI-induced HTR is associated with 5-HT_2A_ receptor activity in several regions of the PFC

DOI-induced HTR in rodents can be blocked by diverse 5-HT_2A_ receptor antagonists [18, 26, 41]. In line with our previous findings [21], currently we show that DOI produces greater frequencies of HTRs at younger age (20- and 30-day old) than in 60-day old ICR mice. Moreover, the selective 5-HT_2A_ receptor antagonist EMD 281014, blocked the evoked HTR in a dose-dependent manner across the age-range tested. In fact, all tested doses of EMD 281014 (0.01, 0.05 and 0.1 mg/kg) significantly reduced the mean frequency of HTR in 20- and 30-day old mice, but larger doses were required to suppress the evoked HTR in 60-day old mice. In line with these findings, published studies in both animals and humans have demonstrated that several 5-HT_2A_ receptor parameters decrease with age, including 5-HT_2A_ receptor number, mRNA, binding affinity, and sensitivity of its signal transduction mechanisms [42–46].

The rodent PFC can be divided into three topologically different regions: the medially located cortical region (mPFC), the ventrally located cortical region (the orbital prefrontal cortex), and the laterally located cortical region [47, 48]. The 5-HT_2A_ receptor is expressed heavily in the PFC and adjacent cortical regions. Almost all prefrontal pyramidal neurons express 5-HT_2A_ receptor, and approximately 20–25% of the glutamic acid decarboxylase-positive interneurons in the PFC express 5-HT_2A_ mRNA [49, 50]. The site of induction of HTR evoked by 5-HT_2A_ receptor agonists appears to be the mPFC since direct infusion of DOI in this region evokes HTRs in rats [19]. Moreover, while 5-HT_2A_ receptor knockout mice are unable to evoke the HTR, genetic expression of 5-HT_2A_ receptor in the cortical neurons of such mice enables DOI produce HTRs [20]. In the present study, DOI-induced c-*fos* expression in different regions at 5 representative rostral-caudal levels of the PFC. In addition, pretreatment with the selective 5-HT_2A_ receptor antagonist EMD 281014 completely prevented the DOI-induced c-*fos* expression in the above PFC regions. Likewise, other 5-HT_2A_ receptor antagonists can block DOI-induced c-*fos* expression in the wild, but not in 5-HT_2A_ knockout mice [15]. Thus, it appears that neuronal circuits in one or more regions of the mice PFC are probably involved in DOI-induced HTR, which are associated with the expression of 5-HT_2A_ receptor in these regions.

### Inhibitory effect of acute administration of MA on DOI-induced HTR might be due to functional interactions between the stimulatory 5-HT_2A_- and inhibitory 5-HT_1A_ receptors

In the current study, we also investigated the effects of acute administration of varying doses of MA (1, 2.5, 5 mg/kg, i.p.) on DOI-evoked HTRs across different ages. As with the discussed 5-HT_2A_ receptor antagonist EMD 281014, MA pretreatment attenuated the mean frequency of DOI-induced HTR in a dose-dependent manner across the age-range tested. In fact, significant reductions occurred in 20- and 30-day old mice by all tested doses of MA, whereas a significant effect was only observed at 5 mg/kg dose of MA in the 60-day old mice. It is interesting to note that larger doses of both MA and EMD 281014 were required to significantly suppress DOI-induced HTR in 60-day old mice. The observed differences in the inhibitory effect of MA or EMD 281014 among different ages of mice probably involve decreased 5-HT_2A_ receptor parameter functions, as well as alterations in 5-HT_1A_ receptor function during aging [42–46]. The inhibitory effect of acute MA administration on DOI-induced HTR might be due to anatomical interconnections as well as functional and neurochemical interactions between 5-HT_2A_ receptor and 5-HT/NE/DA systems. Indeed, such studies reveal that the PFC is innervated by 5-HT, NE, and DA axons from raphe nuclei (RN), locus coeruleus (LC) and ventral tegmental area (VTA), respectively [51–53]. Moreover, the PFC sends projections back to these brainstem nuclei, providing the substrate for feedback control of cortical 5-HT, NE, and DA release [51–53]. In the context of the current study, significant evidence suggests that concomitant activation of either serotonergic 5-HT_1A_- or adrenergic ɑ_2_- receptors, are inhibitory to the induction of 5-HT_2A_-receptor-mediated DOI-induced HTR [27, 28]:

First, the PFC receives dense 5-HT innervation from the RN [54]. In addition, serotonergic 5-HT_1A_ and 5-HT_2A_ receptors are expressed throughout cortical regions especially on pyramidal neurons, 50–60% of which express 5-HT_1A_ and/or 5-HT_2A_ receptors [50]. These two receptors appear to have opposite effects [55]. Indeed, activation of 5-HT_1A_ receptors results in an inhibitory response on the neuronal membrane potential [56, 57], while stimulation of 5-HT_2A_ receptors generates an excitatory response [58, 59].

At the behavioral level, it has been shown that pretreatment with the selective 5-HT_1A_ receptor agonist 8-OHDPAT, attenuates the ability of DOI to induce the HTR [26]. Moreover, the selective 5-HT_1A_ receptor antagonist WAY 100635 was shown to reverse this inhibitory effect [19]. In the current study, the mean frequency of DOI-induced HTR was suppressed by MA in a dose-dependent manner across the age-range tested. Since MA increases extracellular 5-HT levels in the PFC [39], the increased 5-HT can subsequently inhibit DOI-induced HTR via stimulation of 5-HT_1A_ receptors [26]. In order to verify this hypothesis, and since endogenous 5-HT is reportedly involved in the 5-HT_1A_-induced inhibition of the HTR [60], we used a combination of WAY 100635 with MA in the present study. Our results show that pre-injection with WAY 100635 significantly but not completely reversed the inhibitory effect of MA on DOI-induced HTR in 20- and 30-day old mice, indicating that although 5-HT_1A_ receptor plays a major role in the inhibitory effect of MA on DOI-induced HTR, other mechanisms may also be involved. It is generally considered that increased brain levels of 5-HT can potentiate the frequency of HTR via stimulation of 5-HT_2A_ receptors, which may also concomitantly activate the inhibitory 5-HT_1A_ receptors to suppress the maximum frequency of the evoked HTR. Moreover, WAY 100635 behaves as a “silent antagonist” and by itself releases endogenous 5-HT via which evokes the HTR [61]. The above discussed findings further support ability of endogenous 5-HT released by acute administration of MA in suppressing the frequency of DOI-induced HTR via activation of the inhibitory 5-HT_1A_ receptors. However, in the current study, WAY 100635 by itself significantly attenuated the DOI-induced HTR in 20-day old mice but not in 30- or 60-day old mice. This finding indicates that the inhibitory 5-HT_1A_ mechanism is not maximally active at very early age. In addition, another study in older rats has shown that WAY 100635 (0.1 mg/kg; s.c.) can potentiate the HTR induced via bilateral intra-mPFC infusion of DOI (3 µg/0.5 µl/side) but had no effect on basal HTR in control rats [19]. These discrepancies may be due to the differences in injection methods, the time frame of behavioral testing during multiple drug injection protocols, the animals used, and the age-range tested. In addition, our previously discussed study showed that WAY 100635 (0.1–2 mg/kg; i.p.) by itself could evoke HTR in ICR mice within the first 15 min of injection under reversed light/dark condition when tested within the first 5 h of the light cycle but not during the dark cycle [61]. Based on these findings, in the current study, all behavioral experiments were conducted between 9:00 am and 4:00 pm, and the effect of WAY 100635 by itself on basal HTR was not tested.

Second, MA also increases NE levels in the PFC [39] and both adrenergic ɑ_2_- and serotonergic 5-HT_2A_-receptors are enriched in layers I and V in the PFC [50, 62, 63]. While ɑ_2_-adrenergic receptor agonists such as clonidine inhibit the HTR in mice, corresponding antagonists enhance the evoked behavior [64]. In order to clarify whether the ɑ_2_-adrenergic receptor plays a role in the inhibitory effect of MA on the DOI-induced HTR, we used the more selective ɑ_2_-adrenergic receptor antagonist RS 79948 in combination with MA. Inclusion of RS 79948 did not significantly reverse the inhibitory effect of MA on the DOI-induced HTR in 20-, 30- and 60-day old mice. Unlike MA in the current study, we have previously shown that the monoamine reuptake blocker cocaine significantly reduces the frequency of DOI-induced HTR via indirect stimulation of ɑ_2_-receptors through potentiation of synaptic levels of NE [27, 28]. The differences among these studies due to the differences in the pharmacological properties between MA and cocaine, and between RS 79948 and yohimbine. Thus, in the case of inhibitory effects of MA on DOI-mediated HTR, it appears that relative to the impressive suppressive effect of the 5-HT_1A_ receptor, the ɑ_2_-adrenergic receptor did not exert a significant inhibitory role. However, RS 79948 by itself did increase the DOI-induced HTR in 60-day old mice but had no effect on 20- and 30-day old mice, indicating that blockade of ɑ_2_-adrenergic receptor in older mice may enhance the 5-HT dependent HTR, but its mechanism(s) need further investigation. Furthermore, since there is no published evidence that an adrenergic ɑ_2_-adrenergic receptor antagonist can alter DOI-induced HTR, we did not test the effect of RS 79948 alone on basal HTR in each group of mice. Moreover, many of the tested compounds may have additional pharmacological targets, but their effects at specific ages are yet to be studied.

Our corresponding immunohistochemistry data shows that unlike the discussed 5-HT_2A_ receptor antagonist EMD 281014 reducing both DOI-evoked HTRs and c-*fos* immunoreactivity, MA pretreatment only reduced the HTR. Indeed, relative to the vehicle-pretreated control group (vehicle + vehicle), MA potentiated c-*fos* immunoreactivity in several but not all regions of the PFC examined when administered either alone (MA + vehicle) or in combination with DOI (MA + DOI). Furthermore, no additive effect between DOI and MA was observed. Since low doses of MA increases the extracellular levels of all three monoamines in the prefrontal cortex [39], the increase in c-*fos* expression may be due to increased activity of 5-HT, NE and/or DA.

## Conclusion

In summary, the present study confirms that the direct-acting 5-HT_2A/C_ receptor agonist DOI induces the HTR in mice in a dose-dependent manner with older mice evoking fewer HTRs [21]. It further demonstrates that unlike selective serotonin releasers such as d-fenfluramine [23], the currently used nonselective monoamine releaser MA, when administrated acutely, by itself did not evoke the HTR in mice during development across any of the tested ages examined. Both EMD 281014 (0.01–0.1 mg/kg, i.p.) and low doses of MA (1–5 mg/kg, i.p.) attenuated the DOI-induced HTR in a dose-dependent manner across the age-range tested. Unlike MA (5 mg/kg, i.p.), EMD 281014 (0.1 mg/kg) also significantly decreased the expression of DOI-induced c-*fo*s immunoreactivity in several regions of the PFC. The inhibitory effect of acute administration of MA on DOI-induced HTR appears mainly due to functional interactions between the stimulatory 5-HT_2A_- and the inhibitory 5-HT_1A_-receptor via MA-evoked enhancement of synaptic levels of 5-HT. In fact, the 5-HT_1A_ receptor antagonist WAY 100635 significantly reversed the inhibitory effect of MA on DOI-induced HTR in 20- and 30-day old mice, whereas the ɑ_2_ adrenergic-receptor antagonist RS 79948 failed to do so.

## Data Availability

All data are available up on reasonable request from the corresponding author.

## Abbreviations

AI: Agranular insular cortex
Cg1: Cingulate cortex area 1
DA: Dopamine
DAT: DA transporter
DLO: Dorsal lateral orbital cortex
DOI: (±)-2, 5-Dimethoxy-4-iodoamphetamine
DP: Dorsal peduncular cortex
FrA: Frontal associated cortex
HTR: Head-twitch response
IL: Infralimbic cortex
LC: Locus coeruleus
LO: Lateral orbital cortex
M1: Primary motor cortex
M2: Secondary motor cortex
MO: Medial orbital cortex
mPFC: The medial prefrontal cortex
NE: Norepinephrine
NET: NE transporter
PFC: Prefrontal cortex
PrL: Prelimbic cortex
RN: raphe nuclei
S1: Primary somatosensory area
SERT: Serotonin transporter
VMAT-2: Vesicular monoamine transporter-2
VO: Ventral orbital cortex
VTA: Ventral tegmental area
